# Surgical management of Squamous Cell Carcinoma of the lower lip: An experience of 109 cases

**DOI:** 10.4317/medoral.19595

**Published:** 2014-03-08

**Authors:** Wenhao Rena, Yin Lia, Changyang Liua, Cui Qianga, Linmei Zhang, Ling Gaoa, Zhi Wangb, Keqian Zhia

**Affiliations:** 1 MD, DDS, MD, MD Department of Oral and Maxillofacial Surgery, Stomatological Hospital of Xi’an Jiaotong University College of Medicine, Xi’an, Shaanxi, People’s Republic of China.710004; 2 MD, Department of Anesthesiology, Stomatological Hospital of Xi’an Jiaotong University College of Medicine, Xi’an, Shaanxi, People’s Republic of China.710004

## Abstract

Objectives: We are presenting our experience collected from a series of 109 cases with SCC of the lower lip focusing on clinical features of patients and surgical approach. Study Design: We retrospectively analyzed medical records of patients diagnosed with Squamous Cell Carcinoma (SCC) of the lower lip at the Oral and Maxillofacial surgery at Xi’an Jiaotong University during a period between 1999 and 2008. Results: A total of 109 patients with lip cancer were included in the study. When no frozen-section test was performed, the neoplasia was removed with a margin of at least 6 mm. Different surgical techniques were used for lip reconstruction after tumor excision. Neck dissection was performed in all patients with clinically palpable lymph nodes. Median follow-up was 38 months. During follow-up, recurrence occurred in 5 patients, 3 patients developed neck metastases, distant metastases developed in 1 patient. Five patients died during observation period. Conclusions: The patient-related and defect-related issues must be taken into consideration during reconstruction for surgical defect. For N0 patients, we recommend wait-and-see policy. Early detection, careful follow-up and prompt neck is essential for the successful treatment.

** Key words:**Lip cancer, surgical management, reconstruction.

## Introduction

Lip cancer is a common malignancy of the oral cavity and accounts for 23.6% to 30% of malignant tumors of the oral cavity (1-5). The most frequent malignancy related to the lips is squamous cell carcinoma (SCC), while adenocarcinomas and melanoma occur rarely (3,4). The lower lip was the most frequently affected site, which accounts for more than 90% of the cases (2,6).

SCC of the lip is thought to be related to sun exposure. It also explains the greater frequency of the pathologic findings on the lower than on the upper lip, because the upper lip is far less exposed to the sun than the lower (7). However, the etiology of lip cancer is multifactorial, including exposure to sunlight, tobacco, genetic predisposition, immunosuppression and immunodeficiency (7,8). The definitive pathogenic pathway remains unclear.

Surgery is the treatment of choice for most of the tumors of the lip. Surgical resection requires a full-thickness resection of the skin, muscle and underlying mucosa to allow a safe surgical margin. Numerous reconstruction methods after tumor removal have been reported, however, the reconstruction of lip defect remains a challenge (9). Neck dissection should be performed patients with N+ disease, although there is some debate about the measures that should be adopted in N0 cases (1,8,10,11).

In this work we are presenting our experience collected from a series of 109 cases with SCC of the lower lip from January 1999 to April 2008 focusing on clinical features of patients and surgical approach.

## Material and Methods

A retrospective chart review was performed to analyze patients with a diagnosis of SCC of the lower lip treated in the Department of oral and maxillofacial surgery, Xi’an JiaoTong University stomatological hospital, from January 1999 through April 2008.

One hundred and nine patients with preoperative diagnosis of SCC of the lower lip were identified in the medical records within this period. The charts were reviewed, and the following information was recorded: Age and sex, smoking habits and sun exposure, clinical stage, macroscopic features of the primary lesions, type of surgical treatment, follow-up, and outcome.

Analysis of the data was performed using the statistical package software SPSS 13.0 (SPSS Inc., Chicago, IL, USA).

## Results

A total of 109 patients with lip cancer were included in the study. There were 87 men (79.8%) and 22 women (20.2%). The overall male-to-female ratio was 4 to 1. Mean patient age was 62.5 years (range, 32 to 83 years). Distribution of patients according to age is presented in  Table 1. SCC of the lower lip was more frequently found between the ages of 61 and 70 years (45/109, 41.3%). There were 47 patients (43.1%) had the risk factor of sun exposure (farmers), 16 patients (14.7%) smoked more than 10 cigarettes per day.

**Table 1:**
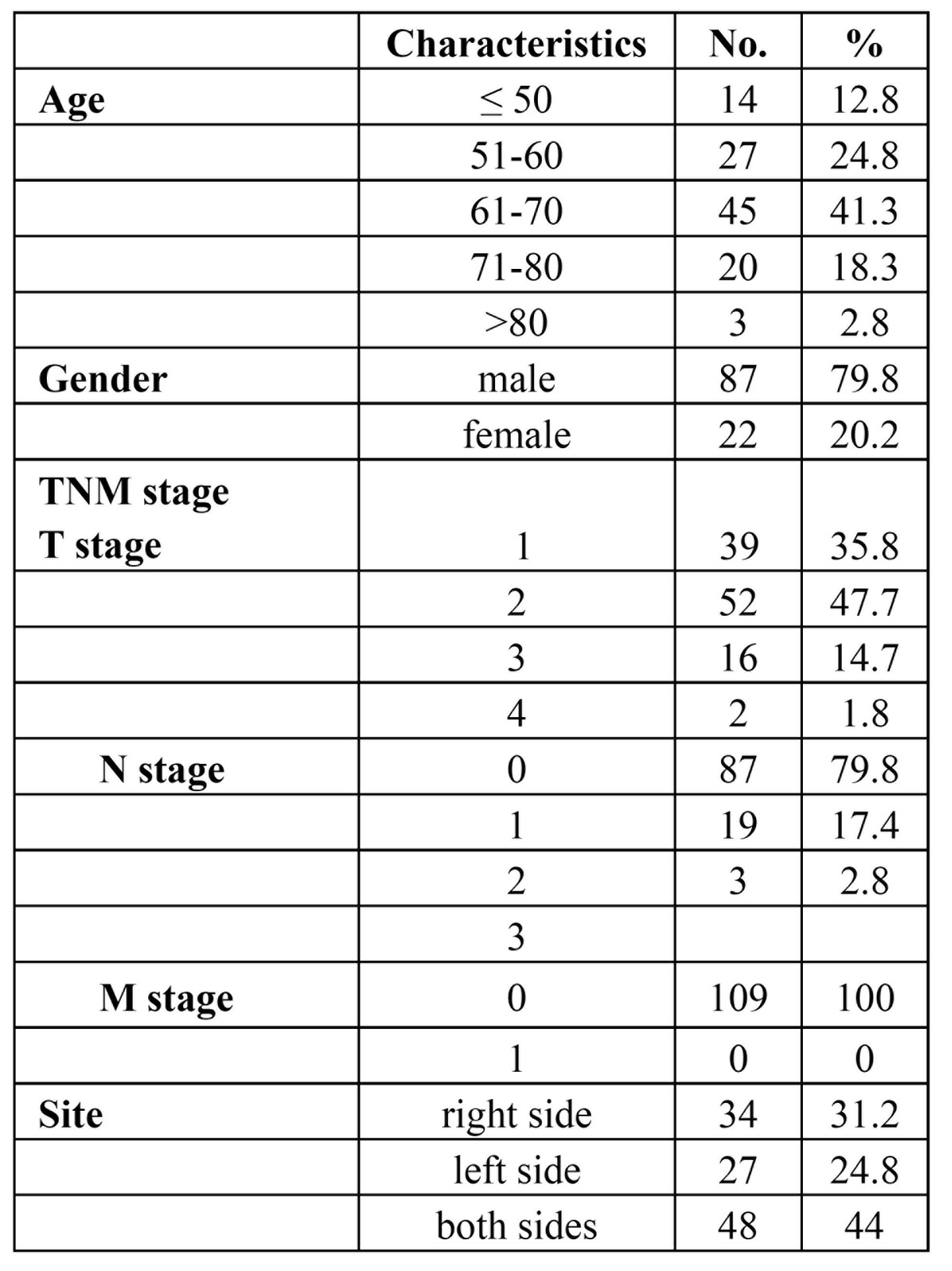
Patient and tumor characteristics.

The right side of the lower lips was involved in 34 patients (31.2%), left side in 27 patients (24.8%), while in 48 patients (44.0%) the tumor involved both sides of the lips.

The distribution of postsurgical TNM stage is shown in  Table 1. There were 39 patients (35.8%) in stage T1, 52 (47.7%) in T2, and 16 patients (14.7%) were in T3 stage. In 2 patients (1.8%) the lesion was in stage T4; 14 patients (12.8%) presented with nodal disease at diagnosis. while in 95 patients (87.2%) there were no signs of clinical regional lymphadenopathy. All patients were in M0. We observed a close relationship between the tumor size at presentation and the neck stage. Two patients with T1-2 disease (2/91) developed lymph node metastases during the follow-up period. In contrast, 6 of 18 patients with T3-4 disease developed lymph node metastases during the study period.

Neck dissection was performed in all patients with clinically palpable lymph nodes (14/109, 12.8%): 3 patients (21.4%) underwent radical neck dissection(RND), 11 patients (78.6%) underwent rational radical neck dissection with conservation of the external jugular vein, deep branches of the cervical plexus, and sternocleidomastoid muscle (12). For the 8 patients who were N0 at diagnosis and who developed lymph node metastasis during the follow-up period, RND were performed in 5, bilateral RND were performed in 2, and radical neck dissection (RND) and contralateral supraomohyoid neck dissection were performed in 1. All the N+ patients were given postoperative radiotherapy.

**Figure 1:**
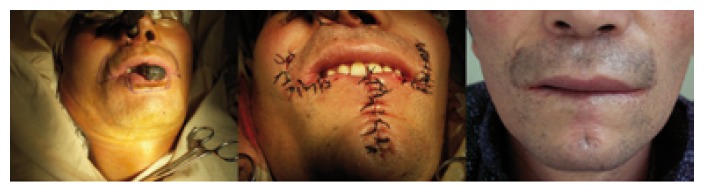
A) Bernard technique for the more than two thirds of the lower lip defect. B) Flap sutured. C) Postoperative result.

**Figure 2:**
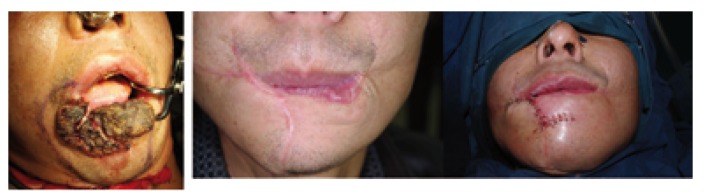
A) Cheek advancement flaps for lower lip reconstruction. B) Postoperative result. C) Scar ectomy and reconstruction of vermilion of the lower lip in the two-stage operation(one year after the first operation).

Different surgical techniques were used for lip reconstruction after tumor excision. For 36 patients (33%) with defects less than one third the length of the lower lip, we used a U or V, W- shape excision. For 56 patients (51.4%) with defects between one third and two thirds lip length of the lower lip, we performed Abbe flap, Bernard flap, Karapandzic flap. For 17 patients (15.6%) with defects more than two thirds lip length of the lower lip, Bernard technique and cheek advancement flaps was used for reconstruction (Figs. 1,2).

Median follow-up was 38 months (range, 12 to 76 months). During follow-up, local recurrence occurred in 5 patients, 3 patients developed neck metastases, 6 of whom were salvaged with surgery and/or radiotherapy. Distant metastases developed in 1 patient. Five patients died during the observation period, 3 of causes unrelated to the disease treated, 2 of the patients died of lip cancer.

## Discussion

Lip cancer is common in malignant neoplasm of the oral cavity. It also has been reported the most common malignant neoplasm of the oral cavity or the second frequency skin cancer in the head and neck region in some studies (8,13). The lower lip was the most frequently affected site. The predilection for this subsite has been attributed to sun exposure, because the lower lip receives considerably more direct sunlight than the upper lip. Sunlight and smoking are substantiated as risk factors for lip cancer development by epidemiological data (14,15). The sex distribution revealed only a slight tendency toward men, which is in contrast to previous studies that reported a male-to-female ratio of 5–8:1 (1,8,10,16).

Several methods for treatment of SCC of the lip are available, including surgery, radiotherapy, chemotherapy, and combinations of these. We preferred surgical removal of the tumor followed by the reconstruction of lip defects for most patients with SCC of the lip. All the patients underwent the surgical treatment in this series. The main goal of the surgery is to completely remove the tumor and the surgical treatment involves full-thickness of the skin, muscle and underlying mucosa to allow a safe surgical margin.

At present, there is no consensus on the deﬁnition of an adequate margin of resection. It has been reported that a 3 mm margin with excision of early SCC of lower lip (stage I/II) seems to be appropriate, when a frozen-section test of the margins is performed (17). If it is not performed, a 6 mm margin is necessary (17). Brodland *et al*. (18) recommended at least a 6 mm margin for high-risk tumors of primary cutaneous squamous cell carcinoma. Some surgeons consider a minimum of 10 mm additional normal tissue at all margins of the resection (16). In this series, when no frozen-section test was performed, we kept a margin of 6-10 mm.

The lips play a key role in facial expression, speech, and eating. The goal of lip reconstruction for the surgical defect should be to maintain oral competence and maximize cosmesis. There are many techniques have been reported for lip reconstruction. However, as a result of the unique anatomy of the lips, reconstruction of the lip can be challenging, especially for the large defect.

Based our experience, a wedge-shaped or V-shaped excision for smaller tumors can be perform for less than one third the length in lower lip defects. If necessary to allow for adequate resection margins, we prefer the U-shaped excisions. They are simple to perform and provide good results.

It may be a challenge for the surgeon on making decision for the defects that are between one third and two thirds the length in low lip. The abbe flap (9), Karapandzic flap (19), Staircase technique (20,21), Gillies fan flap (9,22) and Bernard technique and its modifications (23,24) has been reported to be used for reconstructions involving up to two thirds of the lower lip. According to the size and location of the defect, local tissue characteristics, individual needs of the patient and patient’s condition, we performed local sliding flap, Abbe flap, Bernard technique, Karapandzic flap for 56 patients with defects between one third and two thirds lip length of the lower lip. In our experience, the local sliding flap is easy to performed and suitable to the small tumors; the Abbe flap is used for defects medial to the commissure and could results in a proportional reduction in the size of both the upper and the lower lip, however it need for a two-stage procedure; The Karapandzic ﬂap preserves the nerve and bloody supply but may result in microstomia and distortion of the commissure when used for large defect; Bernard Technique and its several modifications could be used for large defect and the functional and aesthetic result outcome was acceptable. Although not commonly used, the staircase technique has been reported extremely practical and offers good aesthetic and functional results for the up to two thirds defect of the lower lip in several study (1,10,20,21).

For the more than two thirds of the lower lip defect, we used Bernard technique and cheek advancement flaps. Generally, immediate reconstruction after resecting more than two thirds of the lower lip gives poor results. We consider most of these patients need secondary surgery and the result is acceptable. Generally, we don’t recommend the free flap for reconstruction of lower lip, because of the poor appearance and function.

There is no standard or most acceptable surgical technique in lip reconstruction and there was little detailed assessment on postoperative functional and aesthetic outcome for patients following a lip reconstruction in current literature. However, the patient-related and defect-related issues must be taken into consideration during treatment planning.

The reported rate of lymph node metastases at diagnosis is 3% to 29% (1,8,10). In this study, 12.8% of all patients had lymph node involvement at diagnosis. There were 8 N0 cases that became positive during the follow-up period, the percentage with lymph node metastases increased to 20.2%. Many studies have indicated a remarkable worsening of the survival rate for cases that become N+ during follow-up (25). Therefore, how to treat patients with clinical stage N0 disease is in controversy. Some surgeons have adopted an aggressive approach, they suggested that a prophylactic neck dissection should be performed in all N0 cases (11,26,27). Some other authors recommend the “wait-and-see” approach, proceeding to therapeutic RND and radiotherapy, if necessary (8).

We consider it is important to identify the patients who will definitely beneﬁt from an aggressive approach to the lymph nodes. The high risk of lymph node metastases such as tumor size, high histologic grade, locally recurrent, perineural invasion in N0 cases has been reported (4,10,28-30). However, it was found that the tumor size does not correlate closely with regional lymph node metastases in other study (8). In this study, We also observed a close relationship between the tumor size and the neck stage at presentation. Two patients with T1-2 disease (2/91) developed neck metastases during the follow-up period. In contrast, 6 of 18 patients with T3-4 disease developed neck metastases during the study period. JPM Vanderlei *et al*, observed high incidence of neck metastases (more than 20%) in tumors measuring 3 to 4 cm. They suggest that prophylactic neck dissection should be indicated in lip SCCs larger than 3 cm (31){Vanderlei, 2013 #528}.We recommend the ultrasound-guided wait-and-see policy for T1-2 patients with a clinical N0 neck. Certainly compliance of the patient is required.

Early detection and treatment could lead to decreased morbidity and mortality. Therefore, the role of dentists is very important in the early diagnosis of SCC of the lip. Because most of the tumors were diagnosed early (83.5%), SCC of the lip generally has a good outcome. Careful follow-up and prompt neck treatment remain important steps in the surgical management of patients with SCC of the lower lip.

Conclusions

Lower lip cancer is a frequent disease of the oral cavity. General agreement has not been reached concerning in lower lip reconstruction and unresolved questions remain with regard to N0 treatment. We report a series of 109 patients and our experiences on surgical management of SCC of the lower lip. The patient-related and defect-related issues must be taken into consideration during lip reconstruction. For N0 patients, we recommend wait-and-see policy. Careful follow-up and prompt neck treatment remain important steps. Early detection is essential for the successful treatment of SCC of the lip.
